# Computational psychiatry: from synapses to sentience

**DOI:** 10.1038/s41380-022-01743-z

**Published:** 2022-09-02

**Authors:** Karl Friston

**Affiliations:** Wellcome Centre for Human Neuroimaging, Institute of Neurology, University College London, London, WC1N 3AR UK

**Keywords:** Physiology, Biomarkers

## Abstract

This review considers computational psychiatry from a particular viewpoint: namely, a commitment to explaining psychopathology in terms of pathophysiology. It rests on the notion of a *generative model* as underwriting (i) sentient processing in the brain, and (ii) the scientific process in psychiatry. The story starts with a view of the brain—from cognitive and computational neuroscience—as an organ of inference and prediction. This offers a formal description of neuronal message passing, distributed processing and belief propagation in neuronal networks; and how certain kinds of dysconnection lead to aberrant belief updating and false inference. The dysconnections in question can be read as a pernicious synaptopathy that fits comfortably with formal notions of how we—or our brains—encode uncertainty or its complement, *precision*. It then considers how the ensuing process theories are tested empirically, with an emphasis on the computational modelling of neuronal circuits and synaptic gain control that mediates attentional set, active inference, learning and planning. The opportunities afforded by this sort of modelling are considered in light of in silico experiments; namely, computational neuropsychology, computational phenotyping and the promises of a computational nosology for psychiatry. The resulting survey of computational approaches is not scholarly or exhaustive. Rather, its aim is to review a theoretical narrative that is emerging across subdisciplines within psychiatry and empirical scales of investigation. These range from epilepsy research to neurodegenerative disorders; from post-traumatic stress disorder to the management of chronic pain, from schizophrenia to functional medical symptoms.

## Introduction

The inception of computational psychiatry—a decade ago—reflected a consensus that a formal, crisp and clear approach to psychiatry could be furnished by computational advances in data modelling, neuroscience and machine learning [1–5]. There are now annual workshops in computational psychiatry and an eponymous journal. However, introducing computational psychiatry was a risky move: most editors know that that pre-pending any journal title with “Computational” is likely to drop its impact factor by at least one point. So, has computational psychiatry delivered? And where is it going?

Thirty years ago, state-of-the-art computational techniques meant the application of factor analysis to discern patterns of symptoms and signs and schizophrenia—e.g. [6]—or, at least for me, the use of canonical correlation analysis to describe patterns of cerebral blood flow in brain imaging [7]. Nowadays, things are very different. I spend my time discussing the finer details of modelling synaptic efficacy with researchers, whose expertise spans magnetoencephalography, functional genomics, optogenetics and cell cultures. I sit there—pretending to know all the acronyms—and marvel at how a common, computational, narrative can bring molecular biologists and clinical neuroscientists to the same table. This review considers some of the foundational tenets of this narrative, illustrating the ‘work in progress’ with a few select examples.

Our focus will be on computational ideas and procedures that have endured, sometimes for centuries, and are currently [re]emerging across theoretical neurobiology. This limits us to a theory or hypothesis-led approaches, where hypotheses are articulated as a *generative model*, for which empirical evidence can be sought. This demarcates computational approaches with ‘strong’ explanatory power from ‘weak’, descriptive or theory-free approaches. Many ‘weak’ approaches have come and gone, leaving an underwhelming legacy. Examples of weak approaches include behaviourism [8]; namely, a description of behaviour that is reinforced by an outcome that is defined (tautologically) by its ability to reinforce behavior [Clearly, behaviourism is not theory free. I am using ‘behaviourism’ here as a euphemism for simply collecting and summarising dependencies in observable behaviour (e.g., with an autoregression or Rescorla-Wagner model)]. Strong alternatives rest upon the explanatory power of active inference and learning [9], that emphasise beliefs, uncertainty, preferences and curiosity [10–14]. In imaging neuroscience a weak example would be correlation patterns in functional magnetic resonance imaging (fMRI) or electrophysiology, a.k.a., functional connectivity [15]; where its strong counterpart would be the directed connectivity in neuronal circuits that cause the correlations, a.k.a., effective connectivity [16–22]. In the data sciences, weak computational approaches rest on ‘big data’ and machine (e.g., deep) learning procedures that preclude generative or world models and, as a consequence, any mechanistic interpretability or explainability [20, 23, 24]. The strong complement, in machine learning, rests on generative modelling; e.g., generative adversarial networks and automatic variational inference [25–27]. [The bright line between strong (hypothesis-led) and weak (data-led) approaches is, of course, fragile. For example, weak approaches are necessary to furnish evidence for strong approaches (e.g., genome-wide association studies GWAS that point to particular synaptopathies), while hypotheses are necessary to make the best sense of data (e.g., enrichment analysis in prioritizing variants from GWAS)].

The common theme that distinguishes weak from strong approaches is reference to a generative model: namely, a hypothesis about causal mechanisms, specified formally as a joint probability distribution over some (unobservable, latent or hidden) causes and their (observable, measurable) consequences. What follows is, at every level, undergirded by a generative model that places (strong) computational psychiatry—and perhaps our own sentience—firmly in the realm of mechanistic, evidenced-based science; in the sense, that one can compare the evidence for one generative model, relative to another.

## Overview

The first section sets the scene for a mechanistic formulation of psychopathology under current ideas about the brain as an organ of inference, prediction and planning. These ideas can be articulated in terms of the computational processes that accompany belief updating in the Bayesian brain [28–32]. The second section turns to psychopathology—and the underlying pathophysiology—by asking how aberrant belief updating and predictive processing might manifest? In brief, the conclusion is that many psychiatric and neurological disorders can be framed in terms of a synaptopathy that confounds the encoding of uncertainty or precision in a (Bayesian) brain [33–35].

The third section considers the imperatives for computational modelling of empirical data that inherit from the previous sections. This leads to a discussion of how to assess the modulation of synaptic efficacy in terms of changes in effective connectivity, with an emphasis on dynamic causal modelling in psychiatry [20, 36, 37]. The final sections deal with *computational phenotyping*; namely, using computational or in silico models of belief updating—and psychophysical or behavioural concomitants—to explain the responses of a given subject. They briefly consider *computational nosology*, by asking if there is a generative model of pathophysiology and psychopathology that can be used to best explain the trajectories of individual patients using high-density longitudinal data. Finally, the opportunity afforded by a formal and mechanistic understanding of functional brain architectures is exemplified by *computational neuropsychology*; namely, performing in silico lesion or pharmacological experiments.

## The Bayesian brain, precision engineering and active inference

The narrative starts with the simple premise that if psychology—read as belief updating in the brain—can be cast as a computational process of inference, it follows that psychopathology just is false inference. False inference is meant in the usual sense of false positives (i.e., type I errors); namely, inferring something is there when it is not. Cardinal examples here include hallucinations, delusions and other features of reality distortion seen in psychosis. False negatives (i.e., type II errors) mean inferring something is not there when it is; for example, dissociative disorders, neglect syndromes, derealisation phenomena, *et cetera*. Indeed, when one thinks about psychiatric and neurological disorders, most can be framed as false inference: ranging from dysmorphophobia in eating disorders, through to delusional systems in paranoid schizophrenia; from phobias through to Parkinson’s disease. It may seem odd to include Parkinson’s disease; however, the pathognomonic bradykinesia—and failure to initiate movement—can, on one reading, be seen as a failure to realise motor planning as inference (see below). So, what licences the assumption that psychology is inference? [Clearly, asserting that all of psychopathology is ‘false inference’ requires some qualification. The basic argument—that licences this assertion—is that any symptom (or sign) of psychopathology must, at some level, entail aberrant belief updating. The kind of beliefs here are Bayesian beliefs; namely, subpersonal or propositional representations that possess the attribute of uncertainty, and are conditional in a well-specified sense. Under this reading of beliefs, sentient behaviour—and beliefs about that behaviour—can then be cast as inference (e.g., perceptual inference, planning as inference, attribution of agency, emotional recognition, et cetera). Reducing everything to inference then provides a mechanics to understand neuronal dynamics and development, in terms of belief updating and the requisite message in the brain].

The foundations of this account of sentient behaviour can be traced back to the days of Plato and were most clearly articulated in the 19th-century by Helmholtz as unconscious inference [38, 39]—ideas that are reminiscent of Kantian philosophy. These ideas endured through the 20th-century in several flavours; for example, analysis by synthesis [40, 41], epistemological automata [42], perception as hypothesis testing [43, 44] and, in machine learning, the Helmholtz machine [45]. The inference narrative supervened at the turn of the century, with a resurgence of interest in enactivist approaches [46–51] that now predominate in the cognitive and systems neurosciences, in the form of things like predictive processing and active inference [52–60].

Active inference can be read as an enactive version of the Bayesian brain hypothesis [61, 62] that subsumes sentience (perceptual inference) and behaviour by treating control and planning as inference [28–31]. So, what is inference? In this setting, inference just refers to a process that maximises the evidence for some (generative) model or hypothesis about the causes of (sensory) data. Model evidence is also known as marginal likelihood; namely, the likelihood of some data, under a model of how those data were generated. Maximising the evidence for our own generative model is sometimes called self-evidencing [63]. In brief, active inference casts the brain as a fantastic organ: a generator of fantasies, hypotheses and predictions that are tested against sensory evidence. One might ask how this account of sentient behaviour speaks to neuroanatomy, and the functional architectures implied by neurophysiology.

**Fig. 1:**
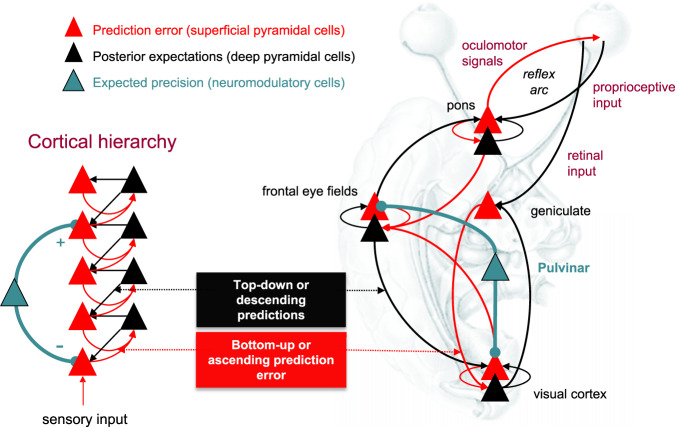
This figure summarizes hierarchical message passing in predictive coding. Predictive coding is a scheme for generative models based upon continuous states. In these schemes, neuronal activity encodes expectations about the causes of sensory input that are continually updated to minimize prediction error. Prediction error is the difference between (ascending) sensory input and (descending) predictions of that input. This minimization rests upon recurrent neuronal interactions among different levels of a cortical hierarchy. It is thought that superficial pyramidal cells (red triangles) compare the expectations (at each hierarchical level) with top-down predictions from deep pyramidal cells (black triangles) of higher levels: see [66] for a review. Left panel: this schematic shows a simple cortical hierarchy with ascending prediction errors and descending predictions. It includes neuromodulatory gating or gain control (teal) of superficial pyramidal cells that determines their relative influence on deep pyramidal cells encoding expectations. Right panel: this provides a schematic example in the visual system: it shows the putative cells of origin of ascending or forward connections that convey prediction errors (red arrows) and descending or backward connections (black arrows) that furnish predictions. The prediction errors are weighted by their expected precision, which have been associated with projections from the pulvinar. In this example, the frontal eye fields send predictions to primary visual cortex, which projects to the lateral geniculate body. However, the frontal eye fields also send proprioceptive predictions to pontine nuclei, which are passed to the oculomotor system to induce movement, through classical oculomotor reflexes. This means that saccadic eye movements, for example, realise top-down proprioceptive predictions that are deeply informed by hierarchical processing of data from all sensory modalities. Similar schemes can be elaborated for discrete state space generative models, responsible for planning and motor control—or allostasis and autonomic function: see [54] for equivalent schematics and [70] for mixtures of continuous and discrete models.

The answer is relatively straightforward. Given a generative model, there are well described belief updating or propagation schemes that specify the requisite message passing that must, in some form, be implemented by neuronal networks. For generative models based upon continuous states of the world, these schemes are known as Bayesian filters or predictive coding [56, 64–67]. In generative models of discrete states (e.g., “I am in the kitchen”, as opposed to “I am at these continuous GPS coordinates”) the message passing schemes are variously known as belief propagation or variational message passing [68–71]. All of these schemes can be cast as a gradient ascent on model evidence or marginal likelihood [72]. In short, neuronal dynamics just are a process of inference. See Fig. 1 for a schematic description of predictive coding.

Crucially, the gradients that subtend neuronal dynamics—and consequent belief updating—can always be formulated as a prediction error [This may sound like a sweeping statement; however, any random dynamical system (including neuronal dynamics) can be cast as a gradient flow, where the gradients can always be expressed as a difference in log probabilities, which can be read as prediction errors of one kind or another] [70]. In other words, the divergence between predictions of sensory input and the observed sensations. In predictive coding schemes, it is thought that prediction errors are represented explicitly: e.g., by superficial pyramidal cells in the upper layers of the cortex [66, 73–76].

This leads to a picture of hierarchical inference in the brain as the reciprocal message passing between the levels of a cortical hierarchy; in which prediction errors ascend from lower to higher levels to drive changes in neuronal populations encoding states of affairs in the world. These populations (e.g., deep pyramidal cells in lower layers of the cortex) then supply a counter stream of descending predictions that resolve or cancel prediction errors at lower levels [66, 75, 77]: e.g., by targeting inhibitory interneurons that are coupled to the superficial pyramidal cells broadcasting prediction errors [78, 79]. This architecture also plays out under discrete generative models and has become something of a workhorse for understanding recurrent message passing in cortical and subcortical hierarchies.

## The importance of being precise

**Fig. 2:**
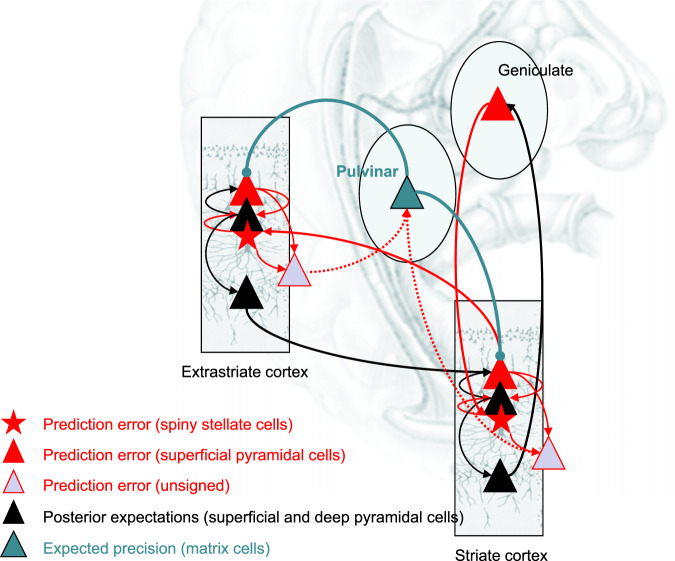
Predicting precision. This schematic is a more detailed version of Fig. 1 that includes putative laminar-specific connections that are consistent with the precision-based predictive coding described in [176]. This architecture is based upon [66] and conforms roughly to the known neuroanatomy and physiology of canonical microcircuits in the visual system and the laminar specificity of extrinsic connections. The key aspect of this figure is the inclusion of deep pyramidal cells encoding the amplitude of (squared or unsigned) prediction error that inform posterior expectations about precision in the (matrix cells) of the pulvinar. These cells reciprocate descending projections to modulate the gain of superficial pyramidal cells in cortex. Forward connections are in red, and descending (backward) connections are in black. First-order prediction errors are shown as full red lines and second-order (unsigned) prediction errors are shown as broken red lines. This schematic ignores inhibitory interneurons that mediate some inferences vicariously. See subsequent figure.

**Fig. 3:**
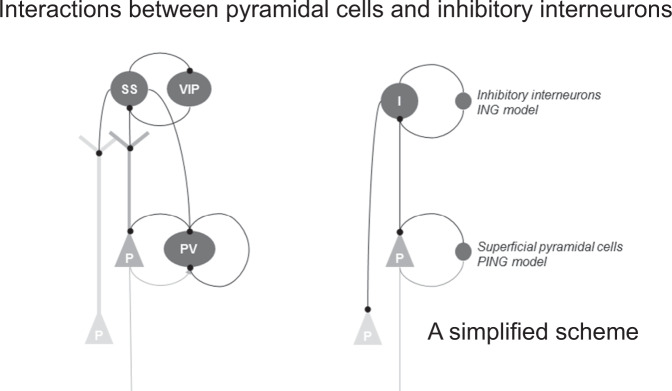
The left panel depicts interactions between (superficial and deep) pyramidal cells with inhibitory interneurons. Inhibitory interneurons are partitioned into three subtypes (Parvalbumin positive PV, somatostatin SST, and vasoactive intestinal peptide expressing interneurons, VIP), based upon optogenetic studies [217]. This schematic illustrates the intimate relationship between pyramidal cells and inhibitory interneurons responsible for excitation-inhibition balance and ensuing excitability in canonical microcircuits. Here, it is assumed that PV interneurons are reciprocally connected to the pyramidal cells through perisomatic compartments, while SST cells form synapses on their dendrites. The right panel shows a simplified architecture used in many modelling studies. Here, the recurrent inhibitory (PV/pyramidal cell) dynamics have been absorbed into an inhibitory recurrent connection, while the SST/VIP interneurons provide (dendritic) inhibitory drive. This allows one to map models of Interneuron Network Gamma (ING) oscillations onto canonical microcircuits used in dynamic causal modeling (see next figure). In this setting, the Pyramidal ING (PING) model emphasizes recurrent interactions among PV cells, as modeled by the inhibitory recurrent connections on superficial pyramidal cells. In contrast, the ING model corresponds to the influence of (SST/VIP) inhibitory interneurons on pyramidal cells. Please see [81] for further details of how these simplified architectures are used to model attentional effects on evoked responses.

Prediction errors (i.e., the gradients that drive belief updating) can be regarded as carrying the newsworthy information at any given hierarchical level to the level above. However, this is not the complete story. Higher levels have to select which prediction errors to listen to; much in the same way that we select our most trustworthy news channels or sources of information. This selection rests upon predictions of predictability or *precision*. Predicting or estimating precision is a universal requisite for making sense of data: from estimating the signal-to-noise ratio in some sensory apparatus, through to estimating standard error when making inferences via some Neyman-Pearson statistic. Affording certain prediction errors greater precision increases their influence on belief updating and has all the hallmarks of attentional selection [80–85]. Physiologically, this simply entails an increase in the excitability or postsynaptic gain of neuronal populations broadcasting prediction errors. On this view, there is an intimate relationship between attention and the modulation of synaptic efficacy by classical neuromodulators and nonlinear postsynaptic responses responsible for mediating the exchange between fast-spiking inhibitory interneurons and (superficial) pyramidal cells [78, 79, 81, 86–91]. Please see Table 1 and [92] for a fuller discussion of neuromodulators and the representation of precision. Figures 2, 3 illustrate the neuronal circuitry implicated in precision or gain control, with a focus on canonical microcircuits and inhibitory interneurons, respectively.

**Table 1:** The pharmacology of precision. Please see [92] for details.

Neurotransmitter system	Functional role	Neuroanatomy
Cholinergic	Encoding the precision of outcomes given hidden states: (c.f., Attention and expected uncertainty [218, 219])	Nucleus basalis of Meynert
Noradrenergic	Encoding the precision of state transitions (c.f., Volatility and unexpected uncertainty [220])	Locus coeruleus
Dopaminergic	Encoding the precision of beliefs about policies (c.f. Action selection [221])	Substantia nigra pars compacta, Ventral tegmental area

One crucial aspect of this precision engineering is that it underwrites our ability to filter out—or ignore—certain prediction errors when they are deemed imprecise. A key example is the attenuation of sensory prediction errors that report the consequences of movement [93–98]. If we could not ignore the proprioceptive and somatosensory afferents—supplying evidence that we are not moving—then any beliefs about intended or predicted movement would be revised immediately, and we would not be able to initiate movement. This mandates a transient suspension of sensory precision during active sensing: c.f., saccadic suppression of optic flow signals during saccadic eye movements [99]. It also provides a glimpse of how one might explain Parkinson’s disease in terms of dopaminergic failures of sensory attenuation [100]. More generally, it foregrounds the role of precision in orchestrating the perception action cycles [101] that underwrite active sensing [102]. For example, sensory attenuation suggests a particular scheduling of action and perception: although both work hand-in-hand to resolve prediction errors, this resolution may involve a fast (~4 Hz) alternation between sampling the world (e.g., visual palpation with saccades) and belief updating after each sample.

In summary, the narrative so far is that psychopathology represents false inference or aberrant belief updating, under a view of the brain as a statistical organ, generating predictions and revising its (subpersonal Bayesian) beliefs on the basis of prediction errors. Crucially, these predictions are contextualised with *predictions of precision* or predictability that instantiate attentional or intentional set; allowing the selection of attenuation of prediction errors via a process of precision weighting. This precision weighting is nothing more than modulating the gain, postsynaptic sensitivity or excitability of appropriate neuronal populations. So, what does this computational architecture offer in terms of potential targets for pathology?

## Synaptopathy, dysconnections and false inference

The narrative returns here to the 19th-century and early formulations of psychiatric disorders such as dementia praecox and schizophrenia. A common theme of these formulations is a disintegration or disruption of neuronal processing. From the perspective of the current narrative, these formulations came in two flavours. First, Wernicke’s sejunction hypothesis [103] emphasised disruption to the organs of connection (i.e., white matter tracts in the brain), positing a form of *disconnection* syndrome [104]. A complementary perspective was provided by Bleuler’s notion of disintegration of the psyche [105], cast in more functional terms that we might now read as a failure of synaptic integration. These ideas re-emerged with the advent of functional brain imaging in the 1990s, in the form of *disconnection* hypotheses, drawing analogies with things like metachromatic leukodystrophy [106] and the *dysconnection* hypothesis [107] that emphasised dysfunctional synaptic integration; specifically, neuromodulatory failures of synaptic function [34]. The latter formulation of dysfunctional connectivity is now arguably the prevalent view for most psychiatric and neurodegenerative conditions, while variants of the sejunction hypothesis would be apt for cerebrovascular accidents, space occupying lesions and other interruptions to white-matter fasciculi: for example, increases in conduction delays due to cerebral small vessel disease in white matter fasciculi.

In short, one could neatly summarise the pathophysiology of many psychiatric and neurological conditions in terms of one or more forms of synaptopathy [108]. Synaptopathy is taken to mean any failure of synaptic function due to a variety pathological mechanisms (i.e., formation, structure, metabolism, etc.). For example, the severe loss of synapse density in human Alzheimer’s disease, frontotemporal dementia, Parkinson’s disease, Progressive Supranuclear Palsy, etc. [109].

But what kind of synaptopathy? A coarse-grained overview of the synaptic theories of schizophrenia suggests that the synaptopathy in question is of a neuromodulatory sort; implicating classical neuromodulatory (ascending) neurotransmitter systems [110–113], GABAergic neurotransmission and NMDA receptors [113–116], where synchronous interactions between fast spiking inhibitory interneurons and pyramidal cells may set the excitability of canonical microcircuits [88, 91, 116–119]. The same story emerges from the functional genomics of schizophrenia, which point to the mechanisms that underwrite neuromodulation, synaptic gain control and ensuing excitation-inhibition balance in neuronal circuits [120, 121]. For example, a recent genome-wide association study [122] of 76,755 individuals with schizophrenia highlights the importance of GRIN2A (which encodes a subunit of the NMDA receptor), while a recent meta-analysis of whole exomes [123] emphasises the role of GRIN2A, as well as GRIA3 (which encodes a subunit of the AMPA receptor). Finally, it is worth noting that nearly all psychoactive drugs, including psychedelics, target classical neuromodulatory or NMDA receptors: e.g., [124, 125].

In short, many psychiatric, neurological and neurodegenerative disorders could be described as arising from pernicious synaptopathies that lead to a functional disintegration of neuronal message passing in cortical and subcortical hierarchies. The particular synaptopathies in question implicate neuromodulatory gain control, of the sort required to deploy precision (i.e., selective attention and sensory attenuation) during inference and planning in the Bayesian brain.

## Too much or too little?

This completes the next step in the narrative; namely, the psychopathology (i.e., false inference) characteristic of psychiatric disorders may be attributable to aberrant precision control, which inherits from synaptopathies that confound neuromodulation. This narrative first emerged in theoretical treatments of hallucinosis in synucleinopathies [126–128] and, from a computational perspective, was developed in schizophrenia [129–133] and autism research [134–136]. Since that time, it has become difficult to find a psychiatric condition that has not been considered through the lens of aberrant precision, in one form or another [21, 135, 137–161].

A crosscutting theme in many of these accounts is a putative failure of sensory attenuation [53, 93–95, 98, 100, 159, 162–165]. In other words, a neuromodulatory failure to attenuate the postsynaptic gain of neuronal populations reporting the consequence of action (in the motor domain or mediated by autonomic reflexes). A failure of sensory attenuation implies an imbalance between the precision afforded sensory prediction errors and the precision of prediction errors deeper in neuronal hierarchies that mediate or maintain prior beliefs of a subpersonal or propositional nature. The ensuing imbalance is often read as a loss of precision or confidence in prior beliefs, relative to the sensory evidence at hand. In autism, this imbalance is consistent with a failure of sensory attenuation and an inability to ignore the sensorium, which may or may not be associated with the neuromodulatory role of oxytocin [135, 166–170].

In schizophrenia, the story is a little more involved; in the sense that a failure of sensory attenuation provides an apt explanation for resistance to illusions (that normally depend upon precise prior beliefs) and failures to elicit mismatch oddball responses (because everything is surprising). Some people then interpret delusional ideation as the brain’s attempt to make sense of unattenuated prediction errors: see also [152, 155, 171, 172]. However, hallucinatory phenomena require a slightly more delicate argument; usually along the lines of a compensatory increase in prior precision that could manifest as a form of paradoxical lesion. In other words, in attempt to override precise sensory prediction errors, higher levels learn to ignore sensory evidence and—sequestered from the sensorium—elaborate false percepts. See [22, 133, 141, 158, 173] for related discussion.

There are many interesting instances of this formulation; ranging from failures of sensory attenuation in autonomic and interoceptive inference, through depression and emotional processing [82, 140, 149, 174], to functional medical symptoms and dissociative phenomena [137, 164]. These computational formulations are nice because they lend themselves to various lines of enquiry; ranging from simulating false inference; e.g., [149], through the careful analysis of psychophysics and choice behaviour e.g., [154, 155, 175]; and, crucially, neurophysiological measurements of synaptic function—to which we now turn.

## Synaptopathy, dysconnections and dynamic causal modelling

The narrative so far is that psychopathology is a pernicious form of false inference that can be attributed to a synaptopathy that confounds the deployment of precision during perceptual inference and ensuing action. This narrative is based upon first principle (i.e., self evidencing and Bayesian) accounts of brain function. Crucially, these accounts have attendant process theories that specify various forms of neuronal dynamics and message passing that can be evaluated empirically [54, 66, 70, 71, 73, 74, 176, 177]. Note that the strong kind of computational psychiatry advocated here demands a process theory, which can be tested empirically, in terms of the evidence for one process theory or model, relative to another.

Although synaptopathy of a certain kind may be a common theme—in terms of computational and pathophysiological mechanisms—where and how these pathologies manifest is an open question: a question that may require a different answer for every condition (and possibly every patient). For example, at the level of (canonical) microcircuits, neuromodulatory abnormalities may manifest in different ways at different levels of cortical hierarchies and, indeed, in the (inter and intralaminar) message passing within microcircuits. This means that one needs a way of identifying functional brain architectures—and, particularly, context sensitive changes in synaptic efficacy—that report the presence or absence of synaptopathy. In short, this calls for in vivo and ex vivo assays of synaptic efficacy [178, 179] that can be deployed across scale; i.e., from molecular biology through to patients in the clinic (or bedside).

There have been amazing advances in the measurement of neuronal processes; ranging from laminar fMRI [180, 181] through to optogenetics [33, 91, 182–184]. From a computational perspective, there is one universal imperative for quantifying changes in synaptic efficacy: one has to be able to assess the evidence—in any empirical measurement—for changes in effective connectivity. In turn, one has to commit to generative models of how the data were generated, in order to assess the evidence for models with and without a change [185]. Again, we come back to the foundational role of generative models but now from the perspective of a scientist, as opposed to the brain (as a scientist). At its simplest, synaptic efficacy is modelled as a connection strength (or rate) with accompanying synaptic rate or time constants. These have to be estimated under some model of hidden or latent physiological fluctuations that generate observed data. This can be at a molecular scale or the scale of whole brain imaging.

In imaging neuroscience, there are two approaches to connectivity; namely the weak approach called *functional* connectivity and the strong approach called *effective* connectivity [186]. Functional connectivity is essentially looking for patterns or correlations between measured neuronal responses. In contrast, effective connectivity is the estimate of directed connection strengths, under some forward or generative model. The power of effective connectivity analyses rests on being able to compare the evidence for one model (sometimes, of functional connectivity as a data feature) relative to another. A prescient example of this is the estimation of intrinsic excitability or gain, usually associated with the neuromodulatory precision control above. To assess the intrinsic (within node or source) excitability of a node—in a distributed network of nodes coupled by extrinsic (between node or source) connections—one has to parameterise network models in terms of intrinsic or self-connections [187]. This foregrounds one of the weaknesses of descriptive or functional connectivity: in the sense that the correlation of a source with itself tells you nothing (it is always one).

In imaging neuroscience, the predominant analysis of effective connectivity is dynamic causal modelling, usually based upon neural mass models with a greater or lesser degree of biological plausibility [16, 17, 19, 20, 22, 36, 37, 188–190]. In brief, modelling neuronal dynamics in terms of neural mass models means that one can parameterise intrinsic and extrinsic connectivity—and context sensitive changes—in terms of model parameters and, crucially, assess the evidence for models with and without certain parameters, by fitting the models to empirical timeseries.

## Measuring synaptic efficacy in vivo

**Fig. 4:**
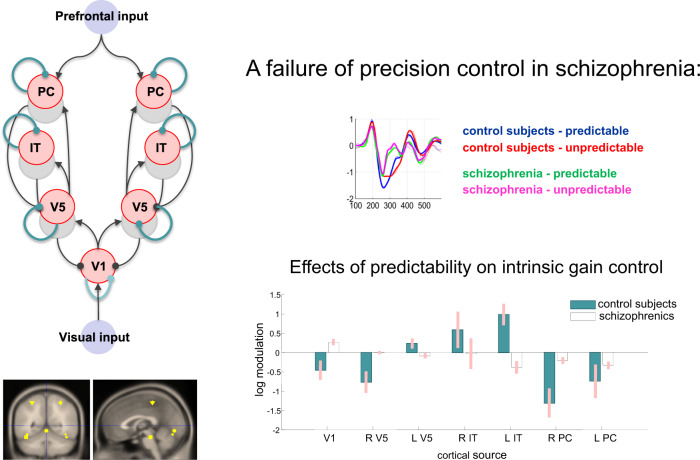
Synaptic gain control in schizophrenia. This figure summarizes an early example of using DCM of event related responses (ERPs), to assess the modulation of synaptic efficacy in schizophrenia using EEG [191]. ERPs were elicited in a visual target detection paradigm, under predictable and unpredictable conditions, in neurotypical and schizophrenic subjects, respectively. Sources—modeled as small cortical patches (lower left panel)—include: a midline visual source (V1), right and left sources near the temporoparietal junction (V5), right and left inferotemporal sources (IT) and bilateral superior parietal sources (PC). The distributed network connecting these sources (upper right panel) entails top-down connections from PC and IT to V5 that send backward connections to V1 (black lines); reciprocal forward connections (red lines); and intrinsic connections for each source (black loops). The principal components of predicted and observed ERPs (upper right panel) show a pronounced difference in the evoked responses of normal subjects to predictable and unpredictable targets around 300 to 400 ms after stimulus onset (compare the red and blue traces). This difference is attenuated in the schizophrenia patients (green and magenta). The lower right panel reports (log scaling of) intrinsic connections and their 90% posterior confidence intervals, for predictable versus unpredictable targets, for patients (white) and controls (teal). The notable thing here is a failure of predictability to modulate the intrinsic gain (i.e., self-connectivity) throughout the hierarchy in the schizophrenic subjects.

Pursuing the narrative of testing hypotheses about neuromodulatory synaptopathies, the three-way link between precision control, attention (and sensory attenuation) and neuromodulation means there has been considerable progress in characterising the synaptic basis of sentience using attentional and oddball paradigms. Indeed, in schizophrenia research, one of the best studied paradigms is the mismatch negativity that allows one to estimate excitability (i.e., intrinsic connectivity) induced by a changing context, signalled by an oddball stimulus; e.g., [22, 191]. Clearly, changes in synaptic efficacy over a hundred milliseconds to seconds requires electrophysiological measurements of the kind afforded by electrophysiology (e.g., EEG and MEG). This allows one to pinpoint the expression of aberrant gain or precision control to various neuronal populations in cortical hierarchies. See Fig. 4 for an early example. The dénouement of this kind of computational study of schizophrenia (at the time of writing) concludes: (i) EEG responses in three classic paradigms are all attributable to the same underlying synaptic change: greater self-inhibition in pyramidal cells; (ii) psychotic symptoms relate to disinhibition in neural circuits and “these findings are more commensurate with the hypothesis that a primary loss of synaptic gain on pyramidal cells is then compensated by interneuron downregulation (rather than the converse)” [192].

**Fig. 5:**
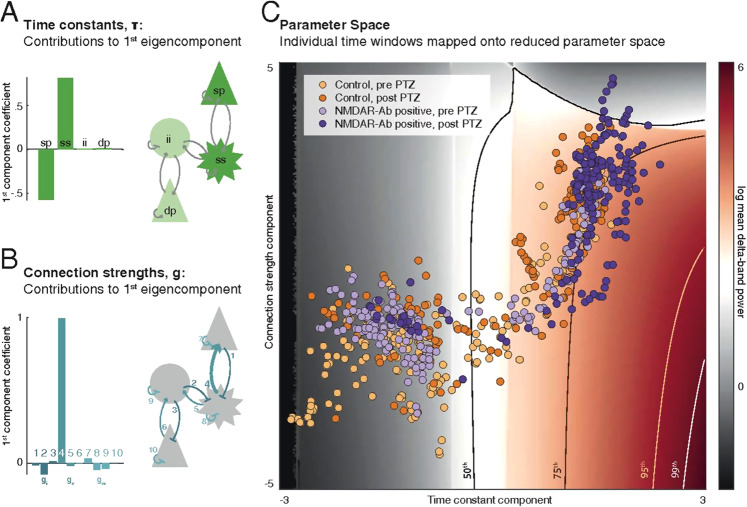
Measuring synaptopathy in vivo. This figure summarises the results of a DCM study of seizure activity in mice—induced with PTZ—in NMDA receptor antibody (Ab) negative and positive cohorts, respectively [197]. Its agenda was to examine the effects of PTZ and receptor antibodies on synaptic efficacy; summarised here in terms of the principal [eigen] component of changes in intrinsic connectivity. The principal component of time constants implicated superficial pyramidal and spiny stellate cell changes (**A**), while the principal component of connectivity-strength reflects changes in the coupling of spiny stellate to superficial pyramidal cells (**B**). The two principal components (of variations in time constants and intrinsic connectivity) constitute a parameter space summarising the modulation of synaptic efficacy. For each point in this parameter space, one can simulate the spectral responses one would observe data space. **C** in this panel, estimates of synaptic efficacy for experimental subjects in the four conditions (pre-and post-PTZ, with and without antibodies) are shown as coloured dots. These are superimposed on the predicted log mean delta power (with selected centile isoclines). The basic message here is that seizure induction with PTZ shifts synaptic efficacy into regimes of high delta (c.f., slow-wave activity), and that this effect is more marked in the presence of NMDA receptor antibodies. dp, deep pyramidal cells; ii, inhibitory interneurons; sp, superficial pyramidal cells; ss, spiny stellate cells. Adapted with permission from the authors from.

The construct validation of these synaptic assays speaks to integration across scales. Perhaps the most developed use cases here are in epilepsy research and the characterisation of neurodegenerative disorders [179, 193–196]. A compelling example of applying generative (i.e., dynamic causal) modelling across scales can be found when drilling down on the molecular basis of childhood epilepsy, using data from small animal models and patients. For example, local field potential recordings in a mouse model were used to validate a dynamic causal model of NMDAR-Ab effects on cortical microcircuitry. The ensuing DCM was then used to identify the synaptic parameters that best explain EEG paroxysms in paediatric patients with NMDAR-Ab encephalitis. The authors then returned to the mouse model to show that NMDAR-Ab-related changes render microcircuitry critically susceptible to overt EEG paroxysms [197]. See Fig. 5.

Another field that is pursuing a similar approach is the characterisation of synaptopathies in neurodegeneration; again, with convergence on the interactions between pyramidal cells and inhibitory interneurons. For example, [198] used the mismatch negativity paradigm to validate a dynamic causal model of laminar-specific microcircuitry and GABAergic neurotransmission. Bayesian model comparison identified an effect of tiagabine on GABAergic modulation of deep pyramidal populations and inhibitory interneurons. The authors then applied this model to patients with frontotemporal lobar degeneration, showing that “the phasic inhibition of deep cortico-cortical pyramidal neurons following tiagabine, but not placebo, was a function of GABA concentration” [199].

Similar approaches have been considered in Parkinson’s disease and schizophrenia, spanning cell cultures to human subjects. The basic idea behind these proposals is to link scales through Bayesian model comparison. In other words, optimise the model of neuronal microcircuits in ex vivo cell cultures, in terms of synaptic time constants of intrinsic connections, using Bayesian model inversion and selection. One can then use the posterior estimates as priors for the next scale (e.g., small animal preparations, through to humans). Crucially, the superordinate scale introduces additional parameters (e.g., extrinsic connections among cortical areas) that are estimated more efficiently, having resolved uncertainty about the parameters that are shared with lower scales. In short, by recursively using yesterday’s (one scale) posteriors as tomorrow’s (next scale) priors, one has a seamless and principled approach to integrating across scales.

## Computational phenotyping and nosology

The narrative so far has moved from the theoretical frameworks, within which to place synaptopathy, to the requisite modelling of empirical data to identify the synaptopathy that characterises different disorders. This could be regarded as one kind of precision medicine [200]; however, there is another kind that speaks to characterising each individual in the sense of personalised medicine. Computational approaches in this setting are potentially important in stratifying and classifying various clinical phenotypes for the mechanistic studies outlined above.

This usually involves summarising a subject’s behaviour in terms of a computational model of that behaviour. Traditionally, this has been a descriptive (e.g., reinforcement learning) model. This represents a weak kind of phenotyping because there are no well-developed process theories for reinforcement learning. A strong complement to computational phenotyping can however be formalised using something called the complete class theorem [201, 202]. This basically says that there for any pair of behaviours and reward functions, there exists some priors that render the behaviour Bayes optimal. This could be read as licensing any Bayesian ‘just so’ stories about choice behaviour [203]. However, it has a deeper and more pragmatic implication.

It means that any given patient can be fully characterised by her prior beliefs under ideal (active Bayesian inference) observer assumptions. This motivates the difficult game of inferring what generative model this subject is using, based purely upon their behavioural or neuronal responses [204–206]. Although at an early stage, this sort of computational phenotyping shows promise, in the sense that it can be more efficient than simply trying to classify or stratify patients on the basis of their responses per se [20]. This approach, sometimes called generative embedding, has been applied both to the generative models a subject might use to specify her behaviour and the generative models the researcher uses to explain her physiological or neuronal responses [4, 207].

A related application of generative models to phenotyping involves constructing dynamic causal models of hidden variables or states that underwrite the signs and symptoms expressed by patients over weeks and years. This was a notable outcome of an Ernst Strüngmann forum on computational psychiatry [208], which concluded that conventional diagnoses—based upon traditional nosology—could play an important role in this kind of computational nosology. However, the role was not to stratify patients but rather as data features that supply evidence for or against models of the mapping between pathophysiology and psychopathology that are hidden from direct observation.

A key tenet of this approach is to cast pathophysiology and psychopathology as slowly fluctuating dynamical processes. This speaks to a separation of temporal scales between processes like active inference [206], active learning [32] and structure learning [209]. The mandatory coupling among these (fast and slow) processes may underwrite cyclothymic disorders, relapsing-remitting presentations and neurodevelopmental determinants. An illustrative example of modelling the dynamics of schizoaffective disorders can be found in [208].

An important aspect—of this application of computational psychiatry—is the quantification of uncertainty about how a patient will behave, and what is likely to happen to her in the future. Indeed, uncertainty quantification is a primary focus of related approaches in quantitative epidemiology and meteorology; especially in the context of forecasting and scenario modelling [210]. In principle, finding the right generative model for a patient guarantees the best predictions. This is because a model with the greatest evidence precludes overfitting and ensures generalisability [211]; namely, the generalisation from old (i.e., what has happened to the patient in the past) to new data (what will happen to her in the future).

## Computational neuropsychology

A final part of the narrative is that a generative model of pathophysiology, and ensuing psychopathology, allows one to perform in silico or synthetic experiments [212–214]. These entail optimising the parameters of a model of a particular subject or cohort, and seeing what would happen if one increased or decreased certain synaptic efficacies, e.g., [197]. Alternatively, one can emulate extremes of aberrant precision by simply deleting connections in active inference models of diagnostic paradigms, e.g., [215]. In these computational studies, the extrinsic (between node) connections are usually assigned to likelihood mappings relating sensory observations to representations or expectations about hidden states of affairs. Conversely, intrinsic (within node) connections are usually treated as embodying prior beliefs about state transitions and narratives that characterise the paradigm in question.

One obvious advantage of being able to create a ‘digital twin’ of a patient in silico, is the ability to perform synthetic lesion studies and psychopharmacology, to test various hypotheses about the effect of therapeutic interventions. This is probably best illustrated in the setting of epilepsy, particularly, in predicting the effects of pharmacological and surgical interventions; e.g., [216].

## Conclusion

This review has taken a somewhat colloquial tour through the fundaments of one (strong) kind of computational psychiatry. Perhaps the take-home message is the foundational importance of a hypothesis or generative model—and being able to articulate this model in computational or formal terms. This is a difficult problem; both in terms of identifying or selecting the right sort of generative models that explain sentient behaviour and its pathologies, but also the biophysical (e.g., dynamic causal) models we, as researchers, use to make sense of our data. This difficulty should not be understated. For example, although dynamic causal modelling has enjoyed wide uptake in fMRI, the requisite models—necessary to explain fine-grained neuronal dynamics in electrophysiology—are notoriously difficult to build and explore using model comparison (a.k.a., structure learning). This usually calls for researchers that are well versed in the system and scale of enquiry, who are also fluent in Bayesian and variational modelling procedures.

The ultimate goal of this endeavour is a generative model or explanation for various psychiatric disorders that furnishes a parsimonious yet accurate account of all the data at hand. This truism reflects the fact that model evidence can be decomposed into accuracy minus complexity; speaking to the pressure to find minimally complex explanations that are as simple as possible but not too simple. In short, the mechanistic explanations we seek will be alluringly simple, but the journey may be difficult. One could argue that psychiatry has chosen the most difficult journey of all; namely, to explain ourselves.

## Glossary

Active inference: securing evidence for a generative model of the world through Bayesian inference and active sampling of (sensory) data. This is equivalent to resolving uncertainty and maximising **model evidence**.
Bayesian belief: a posterior probability distribution over a random variable, such as a latent cause or hidden state of the world causing (sensory) data.
Belief updating: the process of statistical inference, in which Bayes’ theorem is used to update a **prior belief** to a **posterior belief** on the basis of new evidence.
Disconnection (syndrome): a term for neuropsychiatric syndromes caused by damage to the white matter axons of associational or commissural pathways: i.e., a disrupted connection.
Dysconnection (syndrome): a term for neuropsychiatric syndromes caused by a synaptopathy involving associational or commissural pathways: i.e., a dysfunctional connection.
Inference: updating beliefs to maximise **model evidence**. Variational Bayesian inference corresponds to minimising variational free energy, which provides an upper bound on log evidence.
Interoceptive: pertaining to internal (e.g., autonomic) states
Likelihood: the probability of observing some (sensory) data, given the causes of those data
Model evidence: the probability of some (sensory) data under a generative model. Also known as the marginal likelihood, i.e., having marginalised over the causes of the data.
Precision: The inverse variance of a random variable. More generally, the precision of a probability distribution decreases with its entropy.
Prediction: the prediction of some (sensory) data, based upon **posterior beliefs** about how those data were generated.
Prediction error: A quantity used in predictive coding to denote the difference between an observation or point estimate and its predicted value. Predictive coding uses precision weighted prediction errors to update **posterior beliefs** that generate **predictions**.
Prior belief: a belief prior to sampling (sensory) data.
Sejunction hypothesis: (from the Latin noun *seiunctio*: divorce, separation) was introduced by Carl Wernicke (1848-1904) to suggest that psychopathology results from interruption (“sejunction”) of associative connections in the brain.
Variational inference: a tractable form of Bayesian belief updating based upon minimising variational free energy (a.k.a., an evidence bound). The gradients of variational free energy correspond to precision weighted prediction errors.
